# Towards Reliable Adhesive Bonding: A Comprehensive Review of Mechanisms, Defects, and Design Considerations

**DOI:** 10.3390/ma18122724

**Published:** 2025-06-10

**Authors:** Dacho Dachev, Mihalis Kazilas, Giulio Alfano, Sadik Omairey

**Affiliations:** 1 Brunel Composites Centre, College of Engineering, Design and Physical Sciences, Brunel University London, London UB8 3PH, UK; mihalis.kazilas@brunel.ac.uk (M.K.); sadik.omairey@brunel.ac.uk (S.O.); 2 Ford Motor Company, Department of Commercial Vehicles Suspension, Dunton Technical Centre, Basildon SS15 6GB, UK; 3 Department of Mechanical and Aerospace Engineering, College of Engineering, Design and Physical Sciences, Brunel University London, London UB8 3PH, UK; giulio.alfano@brunel.ac.uk

**Keywords:** adhesives, bonding, joining, defects, uncertainties, reliability, environmental degradation, mechanical performance, dissimilar materials, non-destructive testing

## Abstract

Adhesive bonding has emerged as a transformative joining method across multiple industries, offering lightweight, durable, and versatile alternatives to traditional fastening techniques. This review provides a comprehensive exploration of adhesive bonding, from fundamental adhesion mechanisms, mechanical and molecular, to application-specific criteria and the characteristics of common adhesive types. Emphasis is placed on challenges affecting bond quality and longevity, including defects such as kissing bonds, porosity, voids, poor cure, and substrate failures. Critical aspects of surface preparation, bond line thickness, and adhesive ageing under environmental stressors are analysed. Furthermore, this paper highlights the pressing need for sustainable solutions, including the disassembly and recyclability of bonded joints, particularly within the automotive and aerospace sectors. A key insight from this review is the lack of a unified framework to assess defect interaction, stochastic variability, and failure prediction, which is mainly due complexity of multi-defect interactions, the compositional expense of digital simulations, or the difficulty in obtaining sufficient statistical data needed for the stochastic models. This study underscores the necessity for multi-method detection approaches, advanced modelling techniques (i.e., debond-on-demand and bio-based formulations), and future research into defect correlation and sustainable adhesive technologies to improve reliability and support a circular materials economy.

## 1. Introduction

Adhesive bonding has transformed the way materials are joined in various industries, offering a versatile and efficient alternative to traditional mechanical fastening and welding methods. This joining technique involves the application of an adhesive substance that, once cured, forms a strong bond between two or more substrates (the parts being joined). The flexibility of adhesive bonding lies in its ability to join dissimilar materials, distribute stresses evenly across the joint, and reduce the overall weight of assemblies, which is particularly beneficial in sectors such as aerospace, automotive, construction, and electronics.

Advancements in polymer chemistry, surface engineering, and application methodologies have led to adhesives with tailored functional groups, improved toughness, and better environmental resistance, all of which directly contribute to increased bonding reliability and strength. Modern adhesives are engineered to meet specific performance criteria, including high strength, durability, flexibility, and resistance to environmental factors such as temperature extremes, moisture, and chemical exposure. Moreover, the push towards lightweight and fuel-efficient designs in the automotive and aerospace industries has further highlighted the importance of adhesive bonding as a means to enhance structural integrity while minimising weight. A good example of this is seen in some Lotus sports cars, where a combination of bonding and riveting replaces the need for aluminium welding in the Lotus Elise and Evora chassis, which replaced the need for welding (Built-In Lightweight Performance 2017 [1]).

Despite its numerous advantages, adhesive bonding presents several challenges, some are general, such as surface preparation, curing processes, long-term durability under varying environmental conditions, and disassembly and recycling, while others are unique challenges based on operational environments, material combinations, and performance requirements for each industrial sector, as seen in Table 1.

Addressing these challenges requires an understanding of the underlying bonding mechanisms (Section 2), material compatibility, and application techniques (Section 3 and Section 4), and challenges associated with any adhesively bonded joint (Section 6).

This review aims to provide a comprehensive overview of adhesive bonding by exploring its types, applications, characteristics, and the challenges encountered in its implementation. Additionally, emerging trends and future directions in adhesive technology are discussed to highlight potential advancements that could further enhance the efficacy and applicability of adhesive bonds.

## 2. Adhesive Bonding Mechanisms

The ability of adhesive bonding is primarily established by the mechanisms through which adhesion is achieved. A thorough understanding of these mechanisms is essential for selecting the appropriate adhesive and optimising the bonding process for specific applications (Duncan et al., 2003 [2]). These mechanisms can be broadly classified into mechanical and molecular bonding, each contributing uniquely to the overall strength and durability of the bond.

### 2.1. Mechanical Bonding

**Figure 1:**
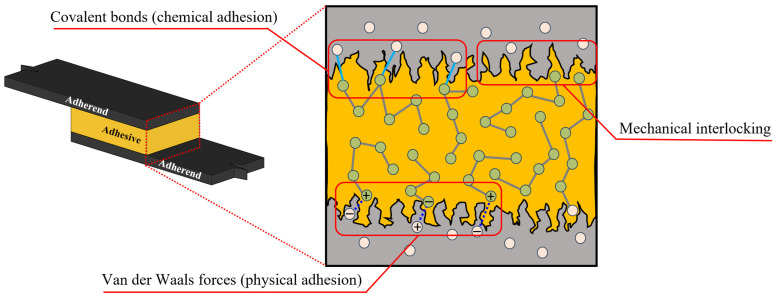
The different categories of bonding mechanisms.

Mechanical bonding relies on the physical interlocking of the adhesive with the substrate’s surface topography; see Figure 1. Surface roughness plays a critical role in enhancing mechanical bonding by increasing the surface area available for adhesion and enabling the adhesive to penetrate the microscopic crevices and asperities of the substrate, creating an interlock (van Dam et al., 2020 [3]). Techniques such as sanding, etching, or abrasive blasting are commonly employed to modify the substrate surface, thereby improving the mechanical interlock and overall bond strength (Trentin et al., 2023 [4]). More details on the substrate surface can be seen in Section 6.5.

The effectiveness of mechanical bonding is influenced by factors such as surface roughness parameters, adhesive viscosity, and the pressure applied during bonding. High-viscosity adhesives are generally better suited for penetrating rough surfaces, whereas low-viscosity adhesives may be preferred for smoother substrates. Nevertheless, it is important to understand the features of bonded surfaces, as high-viscosity adhesives could potentially have limited penetration into very fine micro-crevices or asperities compared to a lower-viscosity adhesives with sufficient wetting time and appropriate surface energy characteristics. Additionally, the uniformity of surface roughness and the presence of contaminants can significantly affect the quality of mechanical bonds (Duncan and Leatherdale, 2005 [5]).

### 2.2. Molecular Bonding

Chemical bonding, as seen in Figure 1, is often referred to as a primary bond (da Silva, Lucas F. M., Öchsner and Adams, 2018 [6]), which involves the formation of molecular-level interactions between the adhesive and the substrate, resulting in covalent, ionic, or hydrogen bonds. Combined with mechanical bonding through intimate contact between the two substrates, this type of adhesion mechanism makes the bond stronger and more durable than mechanical bonding alone, as it provides a connection at the molecular scale (Awaja et al., 2009 [7]).

The strength of chemical bonds is contingent upon the compatibility of the adhesive with the substrate’s surface chemistry. Surface treatments (including a combination of them), such as plasma activation, chemical priming, or the application of coupling agents such as silane, are often necessary to enhance chemical bonding by introducing functional groups that can react with the adhesive (Jung et al., 2023 [8]).

On the other hand, physical bonding is another molecular bonding mechanism (see Figure 1), which is often referred to as secondary bonding (da Silva, Lucas F. M., Öchsner and Adams, 2018 [6]). While physical bonding alone may not provide sufficient strength for structural applications, it plays a significant role in non-structural or temporary bonding scenarios. For example, it contributes to polymer chain stability and is responsible for the physical adsorption of molecules to solid surfaces (Hokkanen and Sillanpää, 2020 [9]).

Physical bonding encompasses Van der Waals forces, which arise from transient dipole moments between molecules (Kuech, 2011 [10]). These interactions are highly dependent on the proximity and orientation of the adhesive and substrate molecules, making physical bonding sensitive to environmental conditions like temperature and humidity (Li, M. et al., 2024 [11]).

## 3. Joining Criteria of Different Applications

**Figure 2:**
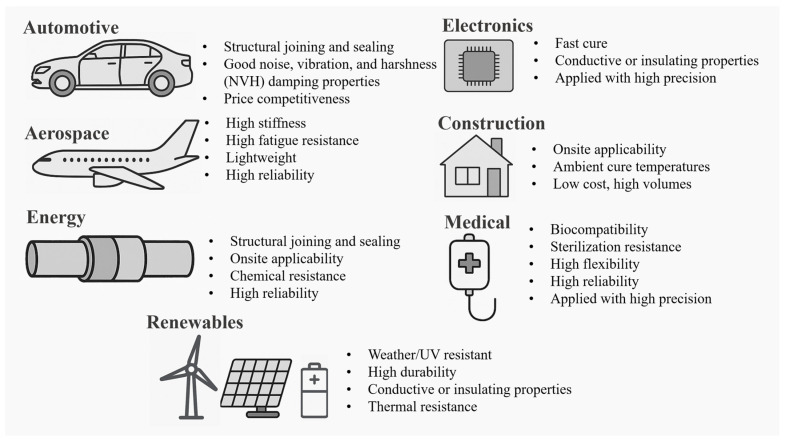
Examples of application-specific requirements for adhesive bonding across key industrial sectors.

Adhesive bonding must be tailored to meet the specific requirements of diverse applications, each with its unique set of performance criteria. The selection of adhesives and bonding techniques is influenced by factors such as the materials to be joined, the operational environment, mechanical load conditions, and the desired lifespan of the bond (Omairey, Jayasree and Kazilas, 2021 [12]). Figure 2 and the following section explains some of these applications and the key bonding criteria associated with them.

In the aerospace industry, for example, adhesive bonds are required to withstand high mechanical stresses, temperature fluctuations, and exposure to harsh environments. Adhesives used in this sector must exhibit high tensile and shear strength, excellent fatigue resistance, and durability, i.e., the ability to maintain performance over extended periods (Ebnesajjad, S., 2009 [13]). Similarly, in the automotive industry, adhesives must accommodate thermal expansion differences between dissimilar materials while also providing resistance to vibration and impact (Sim et al., 2023 [14]). On the other hand, generally, adhesives required for electrical and electronic applications should offer good thermo-mechanical fatigue resistance. However, some electronic applications demand adhesives with specific electrical properties, such as dielectric strength and thermal conductivity, to ensure the reliable performance of electronic components (Nassiet et al., 2021 [15]). These requirements, along with the miniaturised scale and material sensitivity of these applications, limit the scope of available testing. Medical device applications, on the other hand, require biocompatible adhesives that can endure sterilisation processes without compromising bond integrity (Gibbons, 2023 [16]). It is important to highlight that there are limitations and challenges associated with the testing of adhesives in medical applications. For instance, to ensure reliability and ensure that the provided adhesives comply with regulatory requirements and biocompatibility, there is a need to test on human candidates (Mbithi and Worsley, 2023 [17]), which is a challenging process. As a result, this limits the range of viable testing environments and adhesives. Construction applications prioritise adhesives that offer durability, weather resistance, and ease of application to facilitate the assembly of large structures.

### Adhesive Strength vs. Adhesive Characterisation Based on Fracture Mechanics

Within the industry, adhesives are typically characterised in terms of ‘strength’, typically measured in MPa, specifying the test used to measure it, the substrates, and the bonded area. Therefore, the technical data sheet of an adhesive typically provides different values of the adhesive strength for different combinations of substrates, including cases of joints made of dissimilar materials. The most widely used test is the single-lap joint, and the obtained strength, often called ‘lap shear strength’ (Banea, da Silva and Campilho, 2015 [18]; Banea et al., 2018 [19]; Kinloch et al., 2003 [20]) or ‘tensile shear strength’ (Reis, Ferreira and Antunes, 2011 [21]) is obtained by dividing the total force by the bonded area. On the other hand, this value only represents the average shear stress in the joint when the maximum force is reached, which is lower than the maximum stress attained point-wise and depends on many factors including the stiffness of the adherents, possibly the substrate ductility if plastic deformation is induced in the substrates, the adhesive thickness, and the bonded area.

Although the adhesive strength reported in the technical data sheets, as described above, allows engineers in the industry to rapidly compare different products, it is clearly not a property of the material. In other words, the ‘strength’ of the adhesive joint depends on a wide a number of geometrical parameters (including overlap length and adhesive thickness), the elastic and plastic properties of the adherent, and the testing conditions, rather than intrinsic material properties based on more rigorous fracture mechanics principles. Furthermore, in the presence of significant defects, such as cracks due to fatigue, delamination induced by impacts or sufficiently large debonded patches due to partial curing or surface contamination, the failure of adhesives may be mainly driven by their ‘fracture energy’, which for brittle material is related to the adhesive toughness as defined in linear elastic fracture mechanics (LEFM).

Characterisation of adhesives using the concepts of fracture energy can be conducted through different approaches within the general framework of linear or nonlinear fracture mechanics. In this context, the adhesive properties always depend on whether joint failure occurs through a crack propagating in mode I (opening), mode II (shearing), mode III (tearing), or a combination of these (mixed mode). Depending on the ductility of the adhesive, as well as the stiffness of the substrates, a more or less substantial part of the bonded area can undergo progressive damage before full decohesion is reached. This area is called the ‘process zone’ or ‘cohesive zone’, and depending on its size, different approaches are often used. For relatively small cohesive zones, much smaller than the bonded area and characteristic of brittle failure, LEFM is considered valid, and the fracture energy is measured by determining the critical energy release rate, $G_{c}$, which depends again on the failure mode (I, II, III, or mixed). It is widely stated in the literature that cases with larger cohesive zones, comparable to the bonded area, fall outside the range of validity of LEFM, although this is now questionable, as discussed below. In such instances, according to most of the literature, nonlinear fracture mechanics (NLFM) should be employed by replacing $G_{c}$ with the critical value $J_{c}$ of the integral *J*, or, in more extreme cases, one-parameter approaches prove insufficient and more refined formulations are required. In the latter case, cohesive zone models (CZMs) are the most widely used methods.

CZMs introduce a nonlinear relationship between cohesive stresses between the joint substrates and the relative displacements between the two bonded faces at each point of the bonded interface, where each failure mode corresponds to one component of the relevant cohesive stress or displacement. This relationship is often called the traction–separation law (TSL) and is normally characterised by a first part reflecting undamaged behaviour, with high stiffness, followed by a softening part after a peak stress has been reached. Different choices result in different TSLs, the most widely used being bilinear, exponential, and trapezoidal, but many other laws have been used (Alfano, 2006 [22]). The area under the TSL, for each failure mode, represents the so-called ‘work of separation’ Ω.

A large amount of literature spanning several decades covers different methods to characterise adhesive joints based on LEFM, NLFM, and CZMs via analytical and numerical models and with experimental testing. Among the most widely used experimental tests are the double-cantilever beam (DCB) for mode I failure (Škec, Alfano and Jelenić, 2018 [23]) and the end-notched failure (ENF) test for mode II (de Moura, 2006 [24]), whereas the mixed-mode bending test is the most widely used for mixed-mode failure (Liu, Z., Gibson and Newaz, 2002 [25]).

Clear guidance, based on rigorous criteria, on the relative size range of the cohesive zone governing the transition from LEFM to NLFM or CZMs is not available in the literature. In fact, the concept that the cohesive zone size is the actual parameter governing this transition has been proven incorrect by Škec, Alfano, and Jelenić (2018) [23], at least for the case of mode I failure of adhesive joints; their rigorous analysis of the relationship between the energy parameters used in LEFM, NLFM, and CZMs showed that, for a homogeneous interface $\Omega =J_{c}$, the difference between $J_{c}$ and $G_{c}$ is not dependent on the size of the cohesive zone, as widely stated in the literature, but on the change in the energy dissipated in front of the crack tip per unit of new crack area formed. As an important practical implication of this theoretical result, the authors (Škec, Alfano and Jelenić, 2018) [23] showed that LEFM is indeed a valid method for processing DCB tests in the presence of a very large cohesive zone as well, as the inaccuracy entailed is much less than the uncertainties typical for these types of problems. More precisely, the most ductile cases considered by Škec, Alfano, and Jelenić (2018) [23] was a DCB test made of two 300 mm long and 12.7 mm thick flat steel bars bonded with a Sikaforce ^®^ 7752 adhesive, with a fracture energy of 4.5 N/mm and an initial pre-cracked area of 55 mm. For this case, the cohesive zone was found to vary from 45 to 42 mm during the test, which is between 17% and 18% of the bonded area. Yet, the difference between $J_{c}$ and $G_{c}$ is only about 2% at the start of the test and rapidly falls to much less than 1% as the crack propagates.

On the other hand, the above finding by Škec, Alfano, and Jelenić (2018) [23] have been limited to mode I problems and more research should be conducted to extend them to cases of mode II and mixed more, where the contribution of friction can play an important role.

The rate dependence of adhesive properties has also been widely studied using the above methods based on fracture mechanics, due to its importance in many applications in the automotive and aerospace industry, such as in the case of crashworthiness evaluations. A review of some key contributions on this aspect, in terms of both experimental testing and numerical modelling, is provided by Škec and Alfano (2023) [26]. Here, it is worth noting that for different adhesives, different relationships between fracture toughness and crack speed have been found. For example, for the epoxy adhesive Araldite 2015, Škec and Alfano (2023) [26] found an approximate 2.5-fold increase in the fracture energy for a crack speed increasing across five logarithmic decades. For another epoxy adhesive, Betamate XD4600, no significant change in fracture energy was found by Blackman et al. (2009) [27] at low speed, whereas at very high speed, a decrease in fracture energy was reported, which the authors attribute to the effect of heating, due to the transition from an isothermal to an adiabatic process. It is worth noting that Araldite 2015 and Betamate XD4600 are quite different adhesives, the latter being one-component and several times tougher than the former, which is a two-component adhesive. On the other hand, in the authors’ opinion, the main reason for their different rate dependence is the different glass transition temperature, Tg, which is reported to be 127±5 °C for Betamate XD4600 and is much lower for Araldite 2015, in the range between 67 and 89 °C depending on how it is measured and the cure condition.

## 4. Adhesive Types and Dispensing

The vast array of available adhesives can be categorised based on their prevailing method of adhesion, chemical composition, curing mechanisms, and application methods. Selecting the appropriate adhesive type and dispensing method is critical to achieving optimal bond performance and reliability (Duncan et al., 2003 [2]). Some of these commonly used adhesives and their properties are listed in Table 2 below.

In terms of curing mechanisms, whether reactive or non-reactive, adhesives can cure through various mechanisms, including the following:

- Thermal curing: Involves heat-activated crosslinking, suitable for high-strength bonds, although a wide range of adhesives can cure at room temperature (Worrall, Kellar and Vacogne, 2020 [34]);
- UV curing: Utilises ultraviolet light to initiate polymerisation, enabling rapid curing for a wide range of adhesives (The Use of UV Curing in Adhesive Applications 2023 [35]; Worrall, Kellar and Vacogne, 2020 [34]);
- Moisture curing: This relies on ambient moisture to trigger the curing process, commonly used in construction adhesives such as cyanoacrylates, silicones, and polyurethanes (Sánchez-Ferrer et al., 2021 [36]; Worrall, Kellar and Vacogne, 2020 [34]).

In addition to selecting the suitable type of adhesive and cure method, applying the adhesive to the substrate effectively is essential for ensuring uniform bond lines, preventing defects such as voids or air bubbles, and optimising the curing process (Omairey, Jayasree and Kazilas, 2021 [12]). Depending on the type of adhesive, its viscosity, application, and other factors, dispensing methods can be broadly categorised into the following:

- Manual dispensing: Involves the use of syringes, brushes, or rollers, suitable for small-scale or custom applications;
- Automated dispensing: Employs machines and nozzles to apply adhesives with high precision and repeatability, ideal for high-volume production environments;
- Tape and film adhesives: Provide pre-measured amounts of adhesive in tape or film form, simplifying the application process for certain applications. However, they may exhibit limitations in high-precision applications and steering due to their fixed thickness and limited conformability.

In conclusion, the selection of the optimal adhesive type, dispensing method, and cure method must align with the specific requirements of the application, including bond strength, curing time, environmental resistance, level of quality control, and production scalability.

## 5. Fundamental Failure Modes of Adhesively Bonded Joints

**Figure 3:**
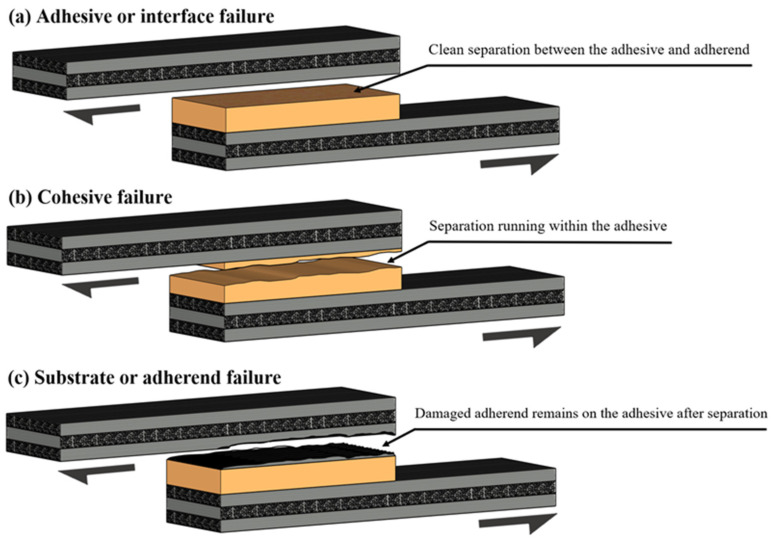
The three main modes of adhesive bond failure: (**a**) Adhesive or interface failure. (**b**) Cohesive failure. (**c**) Substrate or adherend failure.

Before delving into the specific defects and challenges encountered in adhesive bonding, it is essential to first understand the primary failure modes that can occur in adhesively bonded joints. According to ASTM D5573 [37], bond failures can be classified into a range of modes, including adhesive failure, cohesive failure, substrate (adherend) failure, and more complex cases such as fibre tear, light fibre tear, stock break, and mixed-mode failures, where two or more of the aforementioned types occur simultaneously. Among these, three of the most frequently observed failure modes are detailed in Figure 3, which they also represent the most critical forms of failure in structural bonding applications.

### 5.1. Adhesion or Interface Failure

This type of failure is characterised by a lack of adhesion at the interface between the adhesive and one or both adherent surfaces (Davis, M.J. and Bond, 2010 [38]). Typically, no adhesive residue remains on the affected adherend surface, indicating that the bond never properly formed. This can usually, but not always, result from inadequate surface preparation, contamination, or premature curing before establishing full contact. In other cases, even with well-prepared surfaces, other factors can lead to failure in service, which are discussed in more detail in Section 6.6. Operational factors such as cyclic fatigue, adhesive creep, or peel stresses can exacerbate the propagation of this failure mode. In service, such failures often indicate overstressed joints or poor application control (Ren, Chen and Chen, 2017 [39]).

### 5.2. Cohesive Failure

Cohesive failure occurs within the adhesive itself, with adhesive remnants visible on both adherend surfaces (Chadegani and Batra, 2011 [40]). This failure mode typically stems from internal weaknesses, such as voids, poor mixing, or insufficient curing, and is often influenced by joint design, particularly when excessive peel stress or inadequate overlap length is present. Environmental conditions like high humidity or pre-cure ageing of the adhesive can also lead to substandard cohesive strength (Davis, M.J. and Bond, 2010 [38]; Ghorbani, 2018 [41]).

### 5.3. Substrate or Adherend Failure

In this mode, the failure occurs within the adherend rather than the adhesive or the bond line (Hasheminia et al., 2019 [42]). It usually indicates that the adhesive bond is stronger than the substrate material itself. This type of failure is common in thin or brittle materials, such as fibre-reinforced composites or corroded metals. It may also occur due to mismatched material properties or flawed joint design. In laminated composites, the stacking sequence of the plies plays a critical role in how the substrate fails under load.

## 6. Bonding Challenges and Characteristics

Following the understanding of these fundamental failure modes, adhesive, cohesive, and substrate, it becomes evident that many of the defects found in bonded joints originate in, or contribute to, one of these specific failure regions. For example, porosity typically develops within the adhesive layer and can trigger cohesive failure, whereas surface contamination can lead to adhesive interface failure, and micro-cracking or material degradation can result in substrate failure.

**Figure 4:**
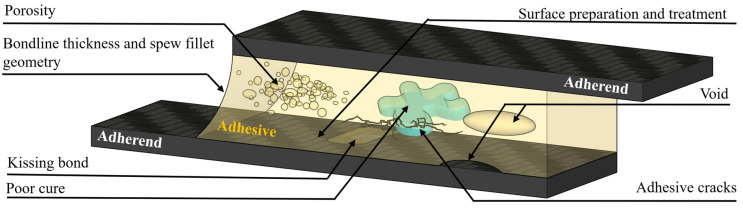
Schematic overview of adhesive joint defects.

To clarify this correlation, Figure 4 presents a schematic overview of an adhesive joint cross-section, identifying the typical locations where key defects such as kissing bonds, voids, porosity, poor cure, and substrate degradation are likely to occur. This figure provides a visual framework that links specific defects with their respective failure zones, setting the context for the more detailed discussion of each defect in the sections that follow.

### 6.1. Kissing Bond

A kissing bond is widely recognised as a defect that results in significant or total loss of adhesion in bonded joints. It is understood that this defect is commonly formed due to surface contamination during the bonding process, leaving sections unbonded. However, other factors can contribute to or create a kissing bond, including incorrect curing processes, inappropriate adhesive composition, and environmental influences. Often, a combination of these factors leads to kissing bond formation.

The thickness of a kissing bond is debated in the literature. Some researchers, such as Vijaya Kumar, Bhat, and Murthy (2013) [43], assert that kissing bonds have zero volume or thickness, while others, for instance, Jiao and Rose (1991) [44], suggest they exist in the nanometre range, smaller than the resolution of many non-destructive testing (NDT) techniques. This makes detection particularly challenging, often rendering conventional NDT methods ineffective. The combination of their critical nature and the difficulty in detecting kissing bonds makes them among the most problematic and dangerous defects in adhesive joints. Kissing bonds can significantly reduce joint strength or lead to premature failure (Vijaya Kumar, Bhat and Murthy, 2013 [43]; Jiao and Rose, 1991 [44]; Steinbild et al., 2019 [45]; Yan, Neild and Drinkwater, 2012 [46]).

This defect is especially important in the aerospace industry, where joint strength and reliability are critical. Consequently, much research has been dedicated to understanding kissing bonds, using both numerical simulations and experimental testing. Initial investigations focused on standard NDT methods, such as ultrasonic techniques. Brotherhood et al. [47] explored various ultrasonic methods, including longitudinal and shear waves, as well as high-power ultrasonic inspection, to evaluate their effectiveness in detecting kissing bonds. The research differentiated between dry and wet kissing bonds, as well as surface roughness, evaluated before bonding. They refer to a dry kissing bond as a result of pure surface preparation, or incomplete cure of the adhesive, where the wet kissing bond is usually a result of residual moisture or some contaminants. Three methods were tested individually and compared under various loading conditions. It was found that high-power ultrasonics were effective at low pressure, but sensitivity diminished as pressure increased. Longitudinal waves provided better contrast in detecting rough surfaces, while shear waves outperformed longitudinal waves on smooth surfaces with medium loads. These results highlight the limitations of individual methods, suggesting that a combination of techniques is necessary for more reliable detection.

To enhance detection capabilities, researchers have explored additional methods. Tighe et al. [48] investigated infrared thermography, specifically Pulsed Thermography (PT) and Pulse Phase Thermography (PPT), alongside ultrasonic C-scan techniques. The study focused on differentiating dry and wet kissing bonds, using contaminants like Frekote, silicon layers, and Polytetrafluoroethylene (PTFE) to simulate defects. The results showed that, while PTFE was not ideal for representing kissing bonds, the liquid layer contact created with contaminants was more realistic. The research also revealed that PPT struggled to detect silicon-induced kissing bonds until a sufficient gap opened under load. PT, on the other hand, required vacuum loading for proper identification. This study reinforced the challenges of detecting kissing bonds and the need for multiple methods to improve detection success.

Due to these challenges, innovative detection techniques have been developed. For example, Bhat and Murthy [43] explored the use of digital image correlation (DIC) to detect dry kissing bonds in glass fibre-reinforced plastic (GFRP) samples. Their results showed that DIC could detect kissing bonds even at 50% of the failure load, with load degradation increasing as the bond area grew. Finite element analysis (FEA) simulations further supported these findings, suggesting that DIC is useful for identifying partial or localised kissing bonds. However, the study noted the need for more tests to establish clear detection boundaries and assess DIC’s feasibility in production settings.

Another innovative approach was explored by Attar et al. (Vijaya Kumar, Bhat and Murthy, 2013 [43]; Attar et al., 2023 [49]), who used Shear Horizontal (SH) guided waves to monitor wave amplitude evolution in samples representing different bond conditions. Their results showed a clear distinction between healthy bonding and kissing bonds, with the signal nearly disappearing in the presence of a kissing bond. While promising, the study lacked clarity regarding detection resolution and the potential for capturing other imperfections.

In addition to detection efforts, researchers have also focused on understanding the mechanical effects of kissing bonds. Jeenjitkaew et al. [50] analysed the impact of various contaminants, including PTFE, Frekote 700-NC, artificial sweat, and cutting oil, on double lap joints using Redux 319 adhesive. Their experiments showed that Frekote caused a significant reduction in shear strength, while artificial sweat and cutting oil had minimal effects. The study also highlighted the migration of contaminants, which influenced the morphology of the bond area and the presence of defects like voids and cavities. The elemental analysis of the Frekote observed the presence of C, O, and Si, which confirmed the presence of polydimethylsiloxane (PDMS), which is one of the most common contaminants found in organic samples. Therefore, PDMS found in Frekote dissolves in volatile organic solvents and dibutyl ether and, therefore, always remains on the substrate after the evaporation of dibutyl ether. Overall, this research provided valuable insights into the correlation between kissing bond formation and mechanical performance.

Further work by Jeenjitkaew et al. [51] investigated the morphology and surface chemistry of kissing bonds using more frequently encountered contaminants. Their study revealed that Frekote left deposits at the interface, with an excess of silica and oxide contributing to defect formation. In contrast, artificial sweat migrated away from the interface, while cutting oil created voids within the adhesive. The mechanical testing results were consistent with previous findings, showing that Frekote-contaminated samples experienced a 27% reduction in strength. The study concluded that Frekote is the most reliable contaminant for recreating kissing bonds, while artificial sweat and cutting oil resulted in less severe defects.

In terms of numerical modelling, Bhat and Murthy [43] employed a cohesive zone approach to model kissing bonds in GFRP adhesive joints. Their FEA simulations, performed using ABAQUS, correlated well with experimental results, providing a useful framework for studying the mechanical effects of kissing bonds. Similarly, Jairaja and Naik [52] modelled weak bonding in single-lap joints between CFRP and aluminium. Their study used different adhesives and introduced weak bonds at various locations within the joint. Like previous research, cohesive zone modelling was used to simulate the kissing bond defects, with all simulations conducted using ABAQUS.

In conclusion, kissing bonds remain one of the most challenging and critical defects in adhesive joints. Despite advancements in detection methods, significant challenges remain. Most research focuses on detecting relatively large areas of kissing bonds, but there is no clear framework defining what defect size is critical or safe. Additionally, while progress has been made in recreating kissing bonds using contaminants, there is still a lack of agreement on how kissing bonds can be reproduced, and a lack of understanding regarding the interaction between multiple contaminants and their effects on defect severity. Finally, the relationship between multiple defects and their combined impact on joint performance remains largely unexplored. Future research should aim to address these gaps, providing a more comprehensive understanding of kissing bonds and their implications for joint integrity.

### 6.2. Porosity and Voids

The literature does not provide a decisive distinction between adhesive porosity and voids; however, generally, porosity refers to clusters of micro-voids within adhesive bonds. As the percentage of voids increases, the effective cross-sectional area of the bond decreases, impacting the structural performance of the joint (Katnam et al., 2011 [53]; Davis, Maxwell, J., 2009 [54]). Porosity is typically caused by volatiles, entrapped air, and the chemical reactions involved in adhesive curing.

Voids, on the other hand, are larger areas devoid of adhesive, with different causes than porosity. Voids here are categorised as a different defect to kissing bonds because they are typically of thickness and can exist anywhere in the bond, not just the interface. Voids can be managed through careful joint design and proper preparation of the adhesive system. They occur due to poor mixing, filling, or laying patterns, or if the joint is disturbed before the adhesive fully cures (Chadegani and Batra, 2011 [40]; Adams, Robert D., 2018 [55]; Katnam et al., 2011 [53]).

Both porosity and voids can be classified as aleatory defects, meaning they occur randomly. More porosity and voids tend to accumulate in the middle of a joint, with fewer near the edges, where air bubbles can escape more easily. Zhang et al. [56] demonstrated the detectability of porosity and voids, as well as a method for artificially generating porosity using PTFE films. The research correlated physical testing and finite element analysis (FEA), showing a linear relationship between failure load and porosity level. Interestingly, as porosity increases, the alignment between physical tests and FEA models improves. The study concluded that while the location of individual voids and porosity is critical to the final performance, it is unlikely that a void near the edge would go undetected and pose a critical risk to the joint.

However, Zhang et al.’s research could be improved by testing more samples to provide a clearer statistical representation and by using more realistic random distributions of porosity. The FEA models, though valuable, exaggerated porosity by representing it as elements spanning the entire adhesive thickness, which does not reflect real-world conditions. No clear threshold for porosity tolerance was identified, and the distinction between porosity and void size remains ambiguous.

Dallali et al. [57] focused on composite joint preparation and behaviour under load, using two adhesives and two preparation techniques. Their research highlighted that flame treatment improved adhesion and bond strength over plasma treatment, which introduced a significant amount of porosity. The study found no direct correlation between initial porosity, glass bead formation, and failure, though further testing is suggested. The study provided little detail on the failure mechanisms, and the lack of post-failure imaging leaves gaps in the conclusions.

Larson et al. [58] evaluated stochastic adhesive porosity and its effects on failure behaviour. The study’s FEA models included porosity and micro-cracks, comparing damaged and pristine joints. Interestingly, some joints with defects outperformed pristine ones, suggesting a progressive failure pattern due to various manufacturing defects. Though porosity was identified as a major factor, it was not deeply explored concerning other defects. Like other studies, porosity was modelled as 2D and spread through the adhesive thickness, which oversimplifies real-world conditions. The absence of acceptance criteria for porosity tolerance limits the study’s practical applicability.

Dumont et al. [59] explored the relationship between porosity and mechanical performance using X-ray microtomography. This research revealed that pores can nucleate and grow during loading, contributing to premature failure. Pores with the most geometric transformation were concentrated in the middle of the adhesive joint, and their distribution followed a normal pattern along the joint’s z-axis. Dumont’s work is valuable, though it lacks a definition of significant vs. non-significant porosity and a clear threshold for porosity tolerance.

Chadegani et al. [40] presented an analytical model to predict void growth under various conditions. Their model captured the onset of void formation and predicted void content based on environmental factors like humidity and temperature. Although the model showed good agreement with experimental results, the assumptions about water saturation and polymerisation simplified the model, resulting in some overestimation of void sizes. There was no stress test to determine when voids become critical nor was there an exploration of void shape and randomness, both of which are important for accurate modelling.

Sengab et al. [60] investigated the interaction between voids and cracks in adhesive joints. Their study found minimal effects of voids on mode I crack growth but significant effects on mode II, especially when voids were close to the crack tip. Flatter, elliptical voids were found to be more detrimental, as they altered the kink angle of the crack. Although Sengab’s research provided useful insights, it focused on a single void size, and further work is needed to explore the impact of multiple, randomly shaped voids on failure mechanisms.

Ref. (Škec and Alfano, 2024) [61] analysed the effect of voids and interfacial failure on the response of double-cantilever beams (DCBs) made of aluminium plates bonded with Araldite 2015 adhesive, by image processing of the fracture surface after their mode I failure. The method allowed for distinguishing between voids and interfacial failure and to evaluate the effective width of adhesive to be used in the DCB analysis. In this way, a more accurate determination of the ‘effective’ fracture energy of the adhesive was determined. Furthermore, the good correlation between experimental data and the results of numerical simulations accounting for interface defects suggests that the typical oscillations of the load–displacement curves experimentally obtained in many adhesive joints tested in a DCB configuration are the result of defects within the adhesives.

In summary, across the literature, porosity is often modelled as a 2D feature that spans the adhesive layer’s thickness, while voids are usually represented as gaps. However, most studies do not account for the randomness of defect distribution or the presence of multiple defects in one joint. Additionally, there is no consensus on the size threshold that differentiates porosity from voids, nor clear acceptance criteria for NDT evaluations. Further research should focus on realistic defect modelling, multi-void scenarios, and defining thresholds for defect significance.

Based on the literature conducted, the authors define porosity as clusters of micro-voids within the adhesive bond and caused by volatiles, entrapped air, and the chemical reactions involved in adhesive curing. On the other hand, voids are larger areas devoid of adhesive. It is believed that they occur due to poor mixing, filling, cure shrinkage, or laying patterns, or if the joint is disturbed before the adhesive fully cures.

### 6.3. Poor Cure and Adhesive Cracks

Poor cure in adhesives refers to a situation where the adhesive fails to fully crosslink or solidify, resulting in reduced strength, flexibility, or durability of the bond. This can be a result of insufficient mix, the use of adhesive beyond pot life, incorrect cure environment, and or other factors. To fully understand the poor curing process in adhesives, several studies must be conducted to analyse factors influencing curing. These factors include ambient conditions, such as temperature, humidity, or lighting, as well as manufacturing variables like batch production variability, adhesive thickness, and chemical composition. Gaining a deeper understanding of these parameters can significantly reduce curing time, enhancing productivity and providing insights into the physical and chemical processes at play during curing (Ford and Tatam, 2012 [62]).

Various cure monitoring techniques are available, such as temperature measurements taken remotely or locally, using infrared detectors or miniature temperature probes embedded in the liquid adhesive. Other methods commonly used include acoustic sensors, dielectric measurement techniques, and optical monitoring (Ford and Tatam, 2012 [62]).

Research by Ford and Tatam [62] has provided valuable insights into the size and detectability of poor cure regions over time during the curing process. They studied samples from a few minutes up to two days of curing time. To simulate a poor mix, epoxy was mixed briefly, and a thin layer (approximately 1 mm) was applied to represent the joint. In their study, the hardener appeared optically clear, while the resin was identified as a scattering area in the correlation images.

### 6.4. Bond Line Thickness

Most of the defects discussed in this section are a direct function of adhesive volume. As bond line thickness increases, the probability of internal defects rises proportionally. This is driven by several factors; for instance, the likelihood of having defects increases, the mixing and application of the adhesive becomes more challenging, and control over cure and exothermic reactions is harder. Additionally, jig stability for joints with thicker bond lines could be compromised. Another effect could result from the stress state thicker joints experience compared with thinner joints, i.e., higher peel stresses relative to shear strength. As early as 1974, Adams and Peppiatt [63] investigated joint strength concerning bond line thickness. It is important to mention that thicker bond lines, generally, although not always (Lopes Fernandes et al., 2019 [64]; Lißner et al., 2019 [65]), resulting in lower joint strength due to the adhesive’s increased plasticity (da Silva, L. F. M. and Campilho, 2015 [66]). Factors such as incorrect machining, misalignment of adherent surfaces, or issues with fixtures can further influence bond line thickness.

Research conducted by Park et al. [67] offers an in-depth analysis of thickness-related issues that align with Adams and Peppiatt’s findings. In their study, single-lap joints were produced with eight different lengths and four varying thicknesses. The failure loads were predicted using damage zone theory, and the theoretical results were compared with experimental data. The stress–strain curve for the adhesive was obtained at the beginning of the tests. During the joint manufacturing process, the researchers emphasised the importance of proper surface treatment and correct manufacturing techniques.

A significant difference in voids and porosity within the adhesive was observed when comparing joints manufactured with and without guide blocks. This difference was noted across three different bond thicknesses. The failure loads for specimens produced with proper manufacturing techniques were 40% and 46% higher than those without, for adhesive lengths of 25 mm and 30 mm, respectively.

During Park’s experimental investigation, joints of various lengths were tested with a bond thickness of 0.15 mm. As expected, it was found that high stress was concentrated at the bond’s ends, while the middle section experienced lower stress, irrespective of adhesive length.

In addition to the above-mentioned examples, where the bond lines are generally thin, up to 1 mm, typical for the aerospace, medical, and electronics industry, there are applications where adhesives are applied in thick bond lines, for instance, in a study conducted by Lahuerta on turbine blades. The study focused on the static and dynamic performance according to certain loading cases, but in many of the cases, there is no investigation on any adhesive defects (Lahuerta, Koorn and Smissaert, 2018 [68]). Furthermore, there is a study conducted by Srinivasan and Vassilopoulos in the field of renewable energy on adhesive layers thicker than 3.75 mm, investigating the defects presented in the layers and some factors affecting the overall quality and performance of the joint (Srinivasan and Vassilopoulos, 2022 [69]).

In conclusion, it was demonstrated that, as adhesive length increases, joint strength decreases. However, the highest failure load across all four thicknesses tested was obtained with a bond thickness of 0.45 mm. The equivalent strain criterion was used to calculate the damage zone when cohesive failure occurred, and the failure loads of joints with different lengths were predicted within 15% accuracy using the damage zone ratio method. When calculating the experiment analytically, the damage zone should be evaluated within the interfacial adhesive layer when using multi-layer elements to model the adhesive. Notably, failure loads obtained through multi-layer adhesive representation were almost identical to those obtained with single-layer adhesive representation. In addition to the above considerations and observations, it is important to note that brittle and ductile adhesives will have different responses to changes in bond line thickness due to their capability to sustain or not sustain different geometrical configurations and how that translates to loading type.

The above research work provides valuable insights into the relationships between bond thickness, adhesive length, and failure loads, demonstrating the impact of factors like stress–strain curves and cohesive failure. The focus on minimising manufacturing defects and improving joint strength is commendable. However, it needs to be mentioned that such results are valid for a specific adhesive and joint configuration described in the research and not conclusive for general joint configurations. While the study addresses manufacturing-related variables and presents effective strategies to reduce defects, there remains potential for further exploration of defect quantification. Incorporating defect metrics into the multi-layer model used in the analysis could enhance understanding of their influence on adhesive strength and provide an even more detailed perspective on failure load predictions.

### 6.5. Surface Preparation and Treatment

As mentioned earlier, common defects such as debonding, cracks, voids, and porosity often result from trapped gases or air during adhesive application, thermal shrinkage, or improper curing. However, as discussed in the section on kissing bonds, defects can also occur due to trapped and contaminated substrates with impurities like oil, grease, or other particles before or during the joining process. Therefore, surface treatment is a critical and highly effective method for enhancing bond strength. The main objectives of surface treatment are to eliminate or minimise oxide layers, contaminants, or other impurities that could lead to poor adhesion (Pragathi et al., 2024 [70]).

As a result of its importance, surface preparation is a highly active area of research, with many research and review papers focusing on the types, advantages, and disadvantages of different surface treatments (Rusen et al., 2024 [71]; Li, X. et al., 2024 [72]; Pragathi et al., 2024 [70]; Liu, J. et al., 2023 [73]; Yudhanto, Alfano and Lubineau, 2021 [74]). For instance, work by Rusen et al. [71] explores an innovative aluminium alloy treatment using the pulsed laser evaporation technique. The study concludes that this method improves stiffness, energy absorption, and maximum shear stress.

Other studies provide an overview of various surface preparation techniques, including their pros and cons, specifically when applied to carbon fibre-reinforced polymers (CFRPs). These papers offer systematic and comprehensive introductions to different surface treatments related to CFRP, concluding that bonding properties are closely related to surface roughness, wettability, and uniform surface morphology (Liu, J. et al., 2023 [73]; Yudhanto, Alfano and Lubineau, 2021 [74]). For instance, a study conducted in 2022 by Hu et al. [75], focuses on pre-coating treatments to create stronger adhesive bonds between titanium alloys and CFRP. This research found that by removing oxide layers and improving surface roughness through a combination of a special resin pre-coating (RPC) and NaOH anodising, bond strength increased compared to grinding and acid pickling alone.

#### Types of Surface Treatments Selected in Groups

The diversity of surface treatments has drawn significant attention, resulting in a wealth of information on the subject. For instance, Critchlow and Brewis [76] identified forty-one different treatments specifically for aluminium alloys. Other studies, such as those of Kanerva et al. (2015a) [77] and Pawlik et al. (2022) [78], have expanded this exploration to include methods for various substrate materials, like composites. In brief, common techniques range from sanding, sandblasting, and solvent cleaning to corona discharge, as well as peel ply, which is frequently used in the aerospace industry for larger surfaces, while the automotive sector prefers more precise techniques like plasma and flame treatments. Furthermore, other innovative approaches such as laser and chemical treatments are steadily gaining popularity.

Given the complexity and variety of these techniques, this review aims to present a comprehensive overview of widely used surface treatment methods. Key characteristics and typical applications of each method are summarised in Table 3.

### 6.6. Adhesive Ageing and Degradation

Adhesively bonded joints are widely used in structural applications, where they are exposed to various environmental factors that can significantly affect joint performance. They can influence the adhesive or the substrate material, often leading to reduced strength, durability, and lifespan. A summary of these environmental elements follows:

- Temperature, a significant factor, can have both detrimental and insidious effects. High temperatures can soften the adhesive, reducing its load capacity, while low temperatures can induce brittleness and cracking, compromising its structural integrity. The cyclical nature of temperature fluctuations, known as thermal cycling, further exacerbates these issues. Repeated expansion and contraction create additional stresses, potentially leading to debonding, micro-cracking, and ultimately, premature failure. It also needs to be mentioned that the glass transition temperature (Tg), which typically exceeds 100 °C for thermosets, can play a significant role in the high-temperature cycle performance mentioned above.
- Excessive sunlight exposure, particularly the ultraviolet (UV) radiation it contains, can cause significant degradation of adhesives, breaking down the polymer chains and leading to embrittlement. This effect is particularly relevant in outdoor applications and can be mitigated through the use of UV-resistant adhesives or protective coatings.
- Moisture also represents a threat to many adhesives, particularly epoxies and polyurethanes, in which it can be readily absorbed from the environment. This absorption can lead to a reduction in strength, stiffness, and an increased susceptibility to failure. Water ingress, the penetration of water into the joint, and hydrolysis, the chemical breakdown of the adhesive by water, can further exacerbate these effects, potentially causing corrosion of the substrate or weakening the adhesive bonds.
- Chemical exposure can also lead to degradation, as contact with oils, fuels, and greases can react with the substrate, resulting in aggressive degradation and weakening of certain adhesives. Pollution, including industrial emissions, dust, and salt, can further negatively impact adhesive bonds, especially in harsh environments like marine applications.
- Other factors such as substrate degradation, cyclic loading and fatigue, atmospheric conditions, pressure, and even biological factors are additional aspects that can influence the long-term performance of adhesive joints.

Several strategies can be employed to mitigate these environmental challenges. Proper adhesive selection, tailored to the specific application and environmental conditions, is crucial. Effective surface preparation, as discussed in the preceding section, plays a critical role in enhancing adhesion and minimising degradation, for instance through water ingress and surface corrosion. Applying protective coatings over the adhesive joint can shield it from harmful environmental factors such as moisture and UV radiation. Incorporating specific additives into the adhesive formulation can further enhance its resistance to degradation.

Research has actively contributed to understanding the degradation mechanism of these environmental factors. For example, Bowditch [96] conducted a comprehensive study exploring the impact of water on the durability of adhesive joints, highlighting the effect of water absorption, particularly in high humidity conditions, leading to a significant reduction in joint strength. This study also emphasises the role of hydrolysis, which can weaken adhesive bonds in systems containing ester and amide linkages, and explores the impact of cyclic fatigue under humid conditions.

Xian et al. [97] investigated the degradation of epoxy adhesives used in underwater bonding applications, examining the effects of freshwater and seawater immersion at various temperatures on the adhesive’s mechanical properties. The study reveals that seawater, due to its higher ionic content, causes more significant degradation compared to freshwater. Elevated temperatures further exacerbate the degradation process, highlighting the critical need for water-resistant adhesives or protective coatings for long-term structural integrity in marine environments.

Korkmaz and Gultekin [98] explored the impact of UV irradiation on the performance of epoxy adhesives, demonstrating that UV exposure can lead to significant degradation in unreinforced epoxy adhesives, reducing their strength and causing embrittlement. However, the study highlights the effectiveness of incorporating boron nitride (BN) and boron carbide (B4C) nanoparticles in enhancing the UV resistance of the adhesive, thereby mitigating the negative effects of UV exposure.

In addition to the studies above, there are also combinations of factors, for example, heat and moisture, also referred to as hygrothermal ageing. This combination of factors is a critical degradation mechanism for epoxy adhesives, causing effects like plasticisation, reduction in glass transition temperature (Tg), potential hydrolysis, swelling stresses, and degradation of the adhesive–adherend interface, ultimately leading to significant reductions in mechanical properties and bond durability. A typical example is provided in a study conducted by Rocha et al. [99], where specimens aged at 50 °C showed a gradual strength decrease with strong correlation with the amount of water absorbed by the specimens. Another study by Odegard and Bandyopadhyay [100] briefly presents observations connected to hygrothermal ageing, connected to weight gain due to moisture intake, increase in micro-crack density and changes in the fracture toughness, and increase in the internal stresses connected to long-term hygrothermal ageing.

These studies provide valuable insights into the complex factors influencing adhesive ageing and degradation. Understanding these factors is crucial for designing robust and durable adhesive joints that can withstand challenging environmental conditions.

Future research should focus on developing novel adhesives with enhanced resistance to specific environmental factors, particularly moisture, UV radiation, and chemical exposure. Investigating the long-term performance of different adhesive systems under realistic environmental conditions and exploring the use of advanced characterisation techniques to better understand the mechanisms of degradation and identify potential failure modes is essential. Developing predictive models to assess the lifespan of adhesive joints under various environmental conditions would also greatly benefit the field. Furthermore, a better understanding of the adhesive’s degradation mechanism could provide accurate information for inspection activities and scheduled maintenance.

### 6.7. The Sustainability Challenge of Adhesive Bonding

The growing emphasis on sustainability within industries like automotive and aerospace is driving the demand for materials and assembly techniques that not only enhance performance but also facilitate end-of-life disassembly and recycling. Adhesive bonding, while offering many benefits such as reduced weight and increased structural integrity, presents a significant challenge when it comes to dismantling bonded components for recycling. Unlike mechanical fastening methods, which allow relatively easy disassembly by removing fasteners, adhesive joints are often permanent and difficult to separate without damaging the adherents. Furthermore, much of the adhesive bonding materials are designed to be chemically stable and, hence, difficult to recycle efficiently. The following sections examine the sustainability aspect of adhesives and recent developments in terms of disassembly and recycling.

#### 6.7.1. Disassembly of Adhesively Bonded Joints

The automotive sector has been at the forefront of adopting advanced materials and joining methods to reduce vehicle weight, improve fuel efficiency, and extend the range of electric vehicles (EVs), aligning with European regulations like the 95 g CO_2_/km target for passenger cars (European Union, 2019 [101]). Adhesive bonding is crucial for using lightweight materials, such as composites and aluminium, in body-in-white (BIW) structures. While these materials offer an excellent strength-to-weight ratio, recyclability challenges arise when adhesives are used.

Currently, separation methods rely on mechanical techniques, such as cutting, which can fracture bonded parts, reducing substrate value. This calls for innovative recycling solutions, like reversible adhesives or selective degradation techniques that preserve the substrate. Physical disassembly methods, such as applying force, thermal energy, or induced defects, can weaken the bond line. From the methods mentioned above, special attention needs to be given to the thermal disassembly. It is important to mention the potential risk of thermal damage to the substrates themselves, especially when dealing with temperature-sensitive materials like polymer composites or certain plastics, which could contradict the benefits of disassembly if the adherends are compromised. Most existing techniques still require an operator and mechanical force to separate substrates (Goodenough et al., 2023 [102]). For example, Broughton’s research [103] suggests using thermally expandable microspheres (TEMs) and expandable graphite (EG) as additives in standard adhesives, allowing controlled bond disassembly through thermal activation that causes expansion and bond separation (Goodenough et al., 2023 [102]).

In addition, new debonding technologies aim to reduce the energy and force needed for adhesive disassembly, supporting substrate reuse and a circular economy. Implementing debonding techniques alongside physical or mechanical methods can significantly lower the required disassembly force, allowing faster and more cost-effective processes. An example of advanced debonding is in Wang et al.’s study [104], which introduces a novel thermo-reversible hot-melt adhesive. This adhesive can bond at low temperatures and release when heated, reverting to a liquid state for easy disassembly, while re-solidifying when cooled. Testing showed minimal reduction in bond strength after repeated bonding–debonding cycles, indicating recyclability and durability. This reversible property suits applications needing temporary but robust bonds, such as electronics or automotive assemblies, where components might need to be dismantled for repair or recycling.

#### 6.7.2. Recyclability of Adhesives

Different adhesives are designed to create strong, durable bonds, made for a purpose, that are challenging to separate. Their widespread use across industries has increased the need for effective recycling methods for both adhesives and substrates at the end of life. Traditional thermal recycling, like incineration, can release harmful chemicals, while mechanical separation often proves ineffective for many adhesives. Consequently, new, innovative recycling solutions are being developed to address this challenge.

Though fully recyclable adhesives remain in development, advancements in materials science and adhesive formulation are paving the way toward sustainable alternatives. Recyclable adhesives have the potential to significantly reduce waste, especially in sectors like packaging and electronics, where adhesives are heavily used.

Bio-based and degradable adhesives represent another solution. Made from renewable resources, like starch or plant oils, these adhesives are designed to degrade naturally with minimal environmental impact. Research by He et al. [105] presents an example of this approach with a bio-based alternative to traditional polyurethane adhesives. Synthesised from resveratrol and epoxidised soybean oil (ESO), this adhesive exhibits high mechanical tensile strength of the joint (up to 48.6 MPa), recyclability, and robust bonding across various materials, including metals, plastics, and wood. The non-isocyanate structure of this adhesive allows it to be broken down and reprocessed, making it suitable for industries like packaging, automotive, and construction, while reducing reliance on petroleum-based products. This study highlights the progress toward eco-friendly alternatives in the adhesive industry, showing that bio-based adhesives can offer both high performance and recyclability. The use of resveratrol and soybean oil enables strong adhesive properties alongside environmental benefits, including easier recycling and a reduced ecological footprint.

Lastly, researchers are exploring new chemical recycling methods that break down adhesives into their basic components (Kim, D. H., Yu and Goh, 2021 [106]; DiPucchio et al., 2023 [107]). Solvent-based processes, for example, can dissolve adhesives, separating them from substrates for reuse. Although still in the early stages, and mostly focusing on epoxies used in composite materials, chemical recycling holds promise for adhesives that are not suitable for mechanical or thermal recycling. More research in this area could unlock additional sustainable recycling options for the adhesive industry.

### 6.8. The Stochastic Challenge of Adhesive Bonding

Despite the significant progress made in characterising defects in adhesive joints and developing non-destructive testing (NDT) methods to detect them, a critical challenge persists in understanding the stochastic, or probabilistic, nature of these defects and their influence on joint performance. Most current approaches to defect analysis and joint design rely on deterministic assumptions. However, this fails to account for the complex and often unpredictable variables that govern the formation, propagation, and interaction of defects in real-world applications.

To enable a more robust and reliability-based design framework, it is crucial to incorporate probabilistic thinking, acknowledging not just the presence of defects but the uncertainty associated with them. This uncertainty can stem from material inconsistencies, environmental conditions, manufacturing variabilities, or combinations thereof. Such a framework could be defined as an integrated approach that combines experimental data, probabilistic modelling, and multi-defect interaction simulations to assess joint reliability in complex loading and environmental conditions. This section highlights several key considerations for advancing the stochastic modelling of adhesively bonded joints:

- Fail-safe design limitations: One of the major hurdles in using adhesives in critical applications (such as aerospace, automotive, or offshore structures) is the difficulty of implementing effective fail-safe features. Certain critical defects, particularly those that are difficult to detect, such as kissing bonds or subsurface porosity, can result in sudden or total loss of bond strength, with little or no warning. This lack of detectability severely limits the effectiveness of traditional safety factors and demands more advanced, reliability-based strategies. Current fail-safe mitigation strategies, such as mechanical fasteners, or Disbond Arrest Features (DAFs), limit the potential of adhesives as they introduce an additional process that leads to high production and maintenance costs, unnecessary weight from the fastener, wider overlapping bond lines to avoid damage caused by drilling defects, and thicker, and hence heavier, substrates to meet thickness requirements.
- Classification of defect uncertainty: As mentioned earlier, defects in adhesive joints can be broadly categorised based on their physical location: at the interface (e.g., poor surface preparation, contamination), within the adhesive layer itself (e.g., voids, porosity, incomplete cure), or in the adherends (e.g., surface cracking, fibre tearing). Each of these defect types can exhibit different kinds of uncertainty:
  **Epistemic** uncertainty arises from a lack of knowledge, such as imprecise material data or limited control over process variables, and can, in principle, be reduced through better testing, monitoring, or modelling.
  **Aleatory** uncertainty, on the other hand, represents inherent randomness, for example, variability in environmental exposure, random bubble entrapment during curing, or uncontrollable contamination, and is much harder to eliminate.
- **Interdependence of defects**: Defects rarely exist in isolation. In practice, many defects interact or are correlated in ways that amplify their effect on joint performance. For example, surface contamination might lead to poor wetting, which in turn increases the likelihood of voids or kissing bonds. To better quantify and predict adhesive joint reliability, it is necessary to evaluate how certain defects influence the occurrence or severity of others.

**Figure 5:**
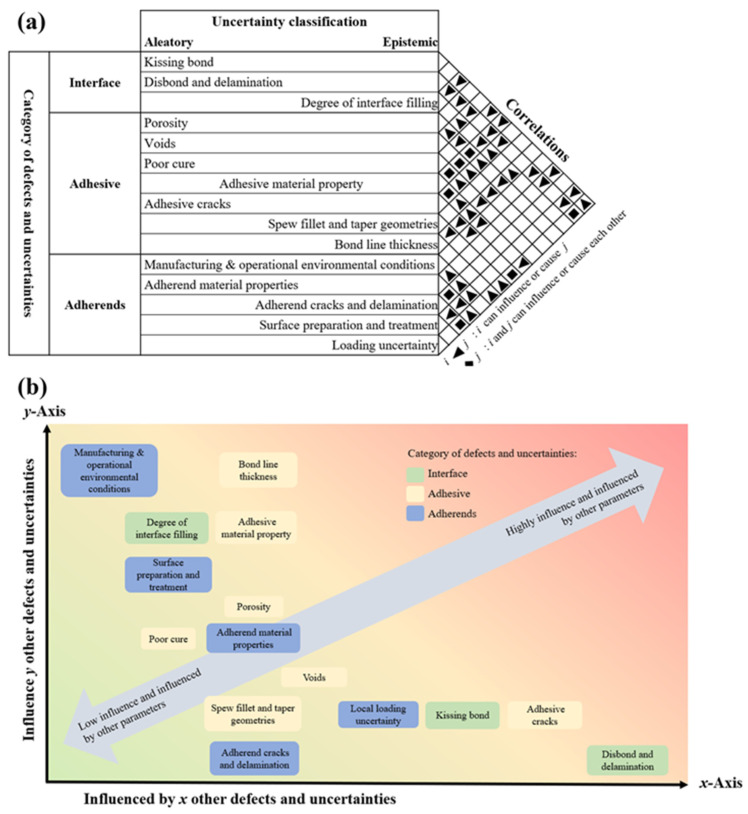
(**a**) Categories, classification, and correlation of common defects and uncertainties within adhesively bonded joints. (**b**) Indicative influence level of the investigated defects and uncertainties.

Based on previous work by the authors (Omairey, Jayasree and Kazilas, 2021 [12]), which relied on an extensive literature review, analysis, and engineering assumptions, this relationship is conceptually illustrated in Figure 5, where the categories, classification, and correlation of common defects and uncertainties within adhesively bonded joints are presented and mapped along two axes: the x-axis reflects how much a defect is influenced by other defects, while the y-axis reflects the extent to which a defect affects other defects or the system as a whole. Defects located higher on the y-axis, such as surface contamination or poor cure, often have a cascading impact and should be prioritised in both modelling and mitigation efforts.

In summary, a shift toward stochastic thinking is essential for the next generation of adhesive bonding research and design. This includes integrating statistical variability into defect models, adopting probabilistic NDT frameworks, and developing simulation tools that can capture the interplay between multiple defect types. By doing so, engineers can move toward more resilient, fail-aware adhesive joint systems, especially in safety-critical or high-reliability applications.

## 7. Conclusions and Recommendations

This review has comprehensively explored the multifaceted domain of adhesive bonding, covering fundamental mechanisms, industrial applications, adhesive chemistries, processing methods, failure modes, and associated defects. It is evident that adhesive bonding plays an increasingly critical role in modern engineering applications due to its versatility in joining dissimilar materials, its capacity to reduce weight, and its potential to distribute stresses more effectively than mechanical fastening.

However, achieving reliable adhesive joints is far from straightforward. A wide range of failure modes, adhesive, cohesive, and substrate-related, can compromise joint performance. These failure modes are often triggered or exacerbated by manufacturing and environmental defects such as porosity, voids, poor cure, surface contamination, and material degradation. This review highlighted that many of these defects originate from or are aggravated by surface preparation inconsistencies, uncontrolled curing conditions, or material incompatibility. Despite advances in adhesive formulations and application techniques, challenges persist in quality control and in reliably detecting critical defects, especially those that remain invisible to conventional non-destructive testing (NDT) methods.

Moreover, the increasingly stringent environmental and sustainability demands placed on industry, particularly in aerospace and automotive sectors, have exposed further limitations of adhesive bonding. The inability to disassemble bonded joints for reuse or recycling remains a significant concern, and while some progress has been made in reversible adhesives and debonding-on-demand technologies, their adoption is still in its infancy.

From a research standpoint, the current landscape shows a fragmented approach to understanding adhesive defects, often addressing individual defect types in isolation. There is a notable lack of integrated studies exploring defect interaction, propagation, and stochastic behaviour under service conditions. Particularly, the interdependence of defects, where one flaw may induce or exacerbate others, has yet to be properly captured in most models. This presents a significant gap in the literature and highlights the need for a paradigm shift toward multi-defect modelling, probabilistic design frameworks, and data-informed reliability assessments.

Looking ahead, several avenues for future research and development emerge:

**Holistic modelling of defects**: There is a need for unified modelling strategies that incorporate both the mechanical and environmental effects of multiple, interacting defects, particularly in relation to their location within the bond line.

**Stochastic and reliability-based design**: Incorporating probabilistic methods to account for material variability, defect distribution, and service loading uncertainties will provide more realistic and safer adhesive joint designs.

**Next-generation NDT techniques**: Continued development of advanced detection methods, such as high-resolution infrared thermography, guided wave ultrasonics, and in situ monitoring tools like digital image correlation, will be essential for detecting nanoscale or subsurface defects like kissing bonds.

**Environmentally conscious adhesives**: Sustainable bonding solutions, including bio-based adhesives, reversible bonds, and recyclable formulations, must move from experimental to applied research phases to meet global circular economy goals.

**Process–material–performance integration**: Future work should aim to link process parameters (surface treatment, curing, dispensing) with resulting material microstructures and long-term joint performance, using tools like multi-scale simulation and machine learning.

In conclusion, adhesive bonding offers immense promise across industries, but its full potential will only be realised through a deeper understanding of the complexities inherent in bond formation and failure. Addressing the challenges highlighted in this review will require interdisciplinary collaboration across materials science, structural engineering, manufacturing, and sustainability research. By embracing integrated, data-driven, and environmentally responsible approaches, the next generation of adhesive technologies can be more reliable, predictable, and aligned with future engineering and environmental demands.

## Figures and Tables

**Table 1:** An overview of industry-specific adhesive bonding challenges.

Sector	Challenges
Aerospace	Extreme temperature: Exposure to cryogenic and high temperatures (i.e., −55 °C to 200 °C) can degrade adhesive performance. Long-term durability: Fatigue, creep, and environmental ageing (UV, humidity) must be accounted for throughout the entire service life. Certification and traceability: Stringent regulatory requirements necessitate full documentation and testing. Bond line inspection: Non-destructive evaluation of bond lines is complex and often limited in reliability.
Automotive	High-volume manufacturing: Adhesives must cure rapidly and be compatible with automation for mass production. Crashworthiness: Bonds must perform reliably under high-strain-rate impacts. Dissimilar material joining: Increasing use of mixed materials (steel, aluminium, composites, etc.) complicates surface treatment and bonding. Thermal cycling: Daily and seasonal temperature fluctuations cause expansion mismatch and fatigue. Recyclability and repair: Adhesives can hinder disassembly and end-of-life recycling.
Wind Energy	Fatigue resistance: Blade bonding must endure millions of fatigue cycles. Environmental exposure: UV, rain erosion, and thermal cycling can degrade adhesive bonds. Large bond areas: Manufacturing defects (voids, mixing, incomplete curing) over large areas and thick bond lines are hard to detect. On-site repairs: Difficulties in carrying out effective in-field repair bonding under variable conditions.
Oil and Gas	Harsh environments: Exposure to high pressure, high temperature, saline water, hydrocarbons, and corrosive chemicals. Long-term reliability: Applications require bonds to last decades without maintenance. Challenging surface preparation: Field applications often face contamination and poor surface preparation conditions. Qualification testing: Highly conservative and rigorous validation is needed for safety-critical systems.
Construction and Infrastructure	Moisture sensitivity: Adhesives used in concrete, glass, and facade bonding are vulnerable to humidity and water ingress. Load-bearing applications: There is limited acceptance for structural applications due to conservative design codes. Temperature and freeze–thaw cycling: These cause expansion–contraction stresses in bonded joints. Long-term creep: Sustained loads can cause slow deformation in adhesives over years.
Marine	Saltwater and biofouling: Accelerates adhesive degradation and surface contamination. Dynamic loading: Vessels and offshore structures experience wave-induced dynamic forces. Surface contamination: Bonding to wet or oily substrates in the field is challenging.
Electronics	Thermal management: Adhesives must maintain thermal conductivity while providing insulation. Miniaturisation: Precise application and curing without damaging components is critical. Outgassing and volatile contaminants: These can affect sensitive electronic circuits. Chemical compatibility: Adhesives must not degrade or corrode adjacent metals or materials.

**Table 2:** Typical types and characteristics of commonly used reactive adhesives.

Adhesive Chemical Composition	General Properties and Application	Key Bonding Substrates	Hardening Time	Most Common Cure Temperature	Key Resistance Features	Cost per Volume
Epoxy	Due to its high mechanical strength, chemical resistance, and excellent bonding capabilities with a wide range of substrates, epoxies are widely used in structural applications (Prolongo, del Rosario and Ureña, 2006 [28]). In many applications, toughened epoxy is used.	Metal Thermoset polymers Range of thermoplastic polymers Glass Concrete Ceramics Wood	Hardens within minutes to an hour and gains full strength within 24 h, depending on the system selected (one or two components) and the temperature used.	60–120 °C	High temperature, water, and impact resistance. Excellent resistance to solvents, mild acids and bases, oil, and fuels.	High
Polyurethane	With a balance of flexibility and toughness, polyurethanes are ideal for applications requiring movement accommodation and impact resistance (Das and Mahanwar, 2020 [29]).	Metal Thermoset polymers Wood	Hardens within half an hour and gains full strength within 6 h, depending on the system selected (one or two components), the temperature used, and the humidity level.	20–60 °C	Good moisture resistance but swell as they harden. Good resistance to oil and fuel, low-concentration acids, but poor resistance to strong solvents.	Low
Acrylic	Known for their rapid curing times and strong adhesion to metals and plastics, acrylics are commonly used in the automotive and construction industries (Dunn, 2015 [30]).	Metal Thermoset polymers Range of thermoplastic polymers Glass Ceramics	Hardens within minutes to an hour and gains full strength within 8 to 48 h, depending on the system selected (one or two components) and the temperature used.	25–80 °C	Resistant to moisture and requires basic surface preparation, such as removing loose materials and surface contaminants. Good resistance to mild acids and solvents, while poor for ketones and chlorinated solvents.	High
Urethane	Urethane adhesives offer a good blend of cohesive strength and flexibility, which makes them very tough, durable adhesives. Urethanes bond well to most unconditioned substrates but may require the use of solvent-based primers to achieve high bond strengths (Sastri, 2022 [31]).	Metal Thermoset polymers Range of thermoplastic polymers Glass Wood	Hardens within minutes to two hours and gains full strength within 6 h to 7 days, depending on the system selected (one or two components) and the temperature used.	25–60 °C	High impact and thermal resistance. Good resistance to moisture, fuel, and oil, while having poor resistance to concentrated solvents and strong acids.	Medium
Cyanoacrylates	Cyanoacrylates provide fast-setting bonds and are suitable for small-scale or quick-repair applications (Ebnesajjad, Sina, 2011 [32]).	Metal Range of thermoplastic polymers Ceramics Wood	Harden in less than a minute and gain full strength within 2 h.	20–25 °C	Limited chemical resistance, can be used for temporary repairs, low impact resistance due to high brittleness, and low moisture resistance.	Very high
Silicone	High thermal stability and flexibility, silicones are used in applications exposed to extreme temperatures and environmental conditions (Han et al., 2022 [33]).	Metal Thermoset polymers Glass Concrete Ceramics Wood	Hardens within an hour and gains full strength within 8 to 24 to 72 h, depending on the system selected (one or two components) and the temperature used.	25–150 °C	High thermal, chemical, and weather resistance. Remains flexible. However, poor resistance to hydrocarbon solvents and strong acids.	Low

**Table 3:** A summary of different groups, types, and details of surface preparation methods, including level of complexity, equipment needed, pros and cons, and indicative cost index.

**Group**	**Type**	Description	Covered Adherent Material	Level of Complexity	Equipment Required	Advantages	Disadvantages	Cost
Mechanical	Sanding/Grinding	Sanding is a mechanical process where the adherent surface is abraded by hand or using a tool, with different types of sandpaper. Usually, the process is divided into two stages, sanding and polishing (Whittingham et al., 2009 [79]; Carnes and Mtenga, 2015 [80]).	Metal Composite polymers Glass Wood	Does not require expensive equipment	Different types of sandpaper and/or a rotating tool	Widely used treatment One of the simplest treatments	Due to manual operation, the surface is not uniform -Post-treatment required -Heavily depends on the operator’s skills	Fairly inexpensive
	Blasting	Blasting, or sandblasting, is a method that uses compressed air as a driving force for small-sized sand particles. Blasting depends mainly on the sand type and size, pressure, angle, speed, and distance. The most used is quartz sand (Hartwig et al., 1996 [81]).	Metals Composite polymers	Complicated and difficult to carry Risk of seriously harming the operator	Require heavy equipment	Used in areas where there are no other alternatives	Depends on many factors -Needs special compressors and scaffolding -Risky to the operator	Usually, a laborious method and costly
Chemical	Peeling	Peeling is a method of removing a ply from the surface of a laminated composite material (Bénard, Fois and Grisel, 2005 [82]; Kanerva et al., 2015 [77]).	Laminated composite polymers		Does not require special equipment	Produces a uniform surface area Can be used over a large area Ensures good repeatability	Removing a ply may create some bumps and pits on the substrate surface -The existence of pits and bumps can generate voids and trap air during the bonding process	Fairly expensive
	Acid etching	This is a chemical reaction, where acid-based reagents react with the surface of the adherent to form some depressions. Treating composites usually requires a bath containing either acid or base solution with specific concentration, temperature, and duration (Hu et al., 2019 [83]; Ebnesajjad, S. and Ebnesajjad, 2013 [84]).	Steel Composite polymers Medical components	Requires very careful handling and safety regulations	Require specific PPE	Can treat large areas	It can be very toxic -Not suitable for small and precise treatments	Fairly expensive
	Solvent cleaning	A solvent is generally a solution that dissolves other substances, but it does not change the chemical composition of the adherent (Zaldivar et al., 2011 [85]; Barthel et al., 2016 [86]).	Composite polymers Plastic Metal	Does not require special equipment or training	Clean cotton clothes	Can treat large and small areas Not harmful Easy to apply Does not cause any damage to the surface	Secondary process -Can leave particles on the surface -An additional process of air blowing is required	Fairly inexpensive
	Anodic oxidation	Also called anodisation, it forms an oxide layer on the surface of the metal, and this film changes the surface state and the properties of the substrate (Takeda et al., 2018 [87]; He, P. et al., 2013 [88]).	Mainly used on metals	Voltage, time, and temperature affect the results of oxidation	Electrolytic system required	Can treat a large area Non-harmful to the operator Environment friendly	Very limited usage, mainly metals	Expensive
High-energy	Laser	This treatment is a form of surface modification of the adherent by the generation of thermal vibration, melting, and vaporisation by the use of a high-energy laser beam (Fischer, Kreling and Dilger, 2012 [89]; Çoban et al., 2019 [90]).	Composite polymers Ceramics Metals	Requires a special level of tuning according to the adherent	Special laser equipment required	Very precise technique Automated process Repeatability Can be used over a large area	Too small power does not remove the contaminants -Too large power can lead to melting and damage to the adherent -Many parameters that reflect on the quality of the process	Expensive
Others	Corona discharge	This is a method that uses the discharge of gas in an uneven electric field, which causes gas ionisation and corona discharge to occur (De Zanet, Salvo and Casalegno, 2022 [91]; Comyn et al., 1996 [92]).	Thermoplastics Ceramic Some metals	Requires precise control of the process parameters	A set of gas nozzles and automated equipment is required	Short process time Fast speed Simple operation and control	Treatment can cause cross-contamination -Can lead to unstable environmental conditions	Expensive
	Plasma	This method uses a thin layer of plasma, which contains active substances that react with the surface of the adherent (Pizzorni, Lertora and Mandolfino, 2020 [93]; Kim, J. K. and Lee, 2002 [94]).	Composites mainly	Requires certain environmental conditions Relatively complicated process, requires control of many parameters	Proper plasma equipment is required	High efficiency Parametric control Environmentally friendly Applicable in a vacuum	Requires low pressure -Fairly new process -Wrong parameter setup can damage the surface of the adherent	Expensive
	Flame	Flame treatment is a relatively new technique that uses burners that work with an air–gas mixture that injects flames, with a temperature of 900–1000 degrees Celsius, through single or multiple nozzles (Adams, R. D., 2021 [95]).	Mainly on composites	Requires some preparations on types of gas used, number of passes, speed, air-to-gas ratio, flow rate, and nozzle-to-surface distance	Require certain equipment: a nozzle set, gas containers, and safety structures	Can be automated Has a fast processing rate and clear control parameters	Reduced speed can promote oxidation. Shear stress drops after treatment Increased duration changes surface morphology	Expensive

## Data Availability

Data sharing is not applicable. No new data were created or analyzed in this study.
